# The Autonomous Fusion Activity of Human Cytomegalovirus Glycoprotein B Is Regulated by Its Carboxy-Terminal Domain

**DOI:** 10.3390/v16091482

**Published:** 2024-09-18

**Authors:** Nina Reuter, Barbara Kropff, Xiaohan Chen, William J. Britt, Heinrich Sticht, Michael Mach, Marco Thomas

**Affiliations:** 1 Institute of Clinical and Molecular Virology, University Hospital Erlangen, Friedrich-Alexander-Universität Erlangen-Nürnberg, 91054 Erlangen, Germany; nina.reuter@uk-erlangen.de (N.R.); barbara.kropff@uk-erlangen.de (B.K.); xiaohan.chen@uk-erlangen.de (X.C.); michael.mach@fau.de (M.M.); 2 Departments of Pediatrics, Microbiology and Neurobiology, Children’s Hospital of Alabama, School of Medicine, University of Alabama, Birmingham, AL 35233-1771, USA; wbritt@uabmc.edu; 3 Division of Bioinformatics, Institute of Biochemistry, Friedrich-Alexander-Universität Erlangen-Nürnberg, 91054 Erlangen, Germany; heinrich.sticht@fau.de

**Keywords:** herpesvirus, human cytomegalovirus, glycoprotein B, gH/gL-independent fusion, cell-cell fusion, blocking fusion, monoclonal antibodies

## Abstract

The human cytomegalovirus (HCMV) glycoprotein B (gB) is the viral fusogen required for entry into cells and for direct cell-to-cell spread of the virus. We have previously demonstrated that the exchange of the carboxy-terminal domain (CTD) of gB for the CTD of the structurally related fusion protein G of the vesicular stomatitis virus (VSV-G) resulted in an intrinsically fusion-active gB variant (gB/VSV-G). In this present study, we employed a dual split protein (DSP)-based cell fusion assay to further characterize the determinants of fusion activity in the CTD of gB. We generated a comprehensive library of gB CTD truncation mutants and identified two mutants, gB-787 and gB-807, which were fusion-competent and induced the formation of multinucleated cell syncytia in the absence of other HCMV proteins. Structural modeling coupled with site-directed mutagenesis revealed that gB fusion activity is primarily mediated by the CTD helix 2, and secondarily by the recruitment of cellular SH2/WW-domain-containing proteins. The fusion activity of gB-807 was inhibited by gB-specific monoclonal antibodies (MAbs) targeting the antigenic domains AD-1 to AD-5 within the ectodomain and not restricted to MAbs directed against AD-4 and AD-5 as observed for gB/VSV-G. This finding suggested a differential regulation of the fusion-active conformational state of both gB variants. Collectively, our findings underscore a pivotal role of the CTD in regulating the fusogenicity of HCMV gB, with important implications for understanding the conformations of gB that facilitate membrane fusion, including antigenic structures that could be targeted by antibodies to block this essential step in HCMV infection.

## 1. Introduction

Human cytomegalovirus (HCMV) is a highly prevalent betaherpesvirus that poses significant health risks to immunocompromised individuals and neonates infected in utero. HCMV is the leading cause of congenital viral infections, affecting one in one hundred and fifty live-born infants globally [1]. HCMV is also the primary infectious agent causing sensorineural hearing loss in children and is linked to various neurodevelopmental disorders [2]. In stem cell or solid organ transplant recipients, HCMV infection significantly increases morbidity and mortality rates [3,4]. Utilization of currently available antiviral therapies is limited by their toxicity and the emergence of drug-resistant viral strains [5]. Consequently, prophylactic vaccination is proposed as the most effective strategy to prevent HCMV infection and its associated complications [6].

Glycoprotein B (gB) has been identified as a promising candidate for HCMV vaccine development secondary to its role as the viral fusogen, facilitating both entry into cells and direct cell-to-cell transmission [7]. gB is essential for viral replication and is highly immunogenic, with gB-specific antibodies being detectable in all individuals with natural HCMV infection [8]. A substantial fraction of virus-neutralizing antibodies in the sera of HCMV-seropositive individuals targets gB with the overall neutralizing capacity of immune serum correlating with anti-gB antibody titers [9,10]. Owing to the potent and broad neutralization by anti-gB antibodies across different viral isolates, gB is among the targets selected for HCMV vaccine development. In fact, the most successful HCMV vaccine to date is a recombinant glycoprotein B (gB) subunit vaccine formulated with the MF59 adjuvant. In a phase 2 trial involving postpartum women, this vaccine demonstrated approximately 50% efficacy in preventing HCMV acquisition [11]. Additionally, it provided up to 45% efficacy in preventing HCMV infection in seronegative females [12]. This vaccine also showed protective effects against HCMV viremia and reduced the need for antiviral treatment in transplant recipients [13]. The partial protection observed in clinical trials may be due to the gB/MF59 vaccine’s inability to elicit robust neutralizing antibody responses [14,15,16]. Additionally, the antibody profile generated by the gB/MF59 vaccine differs significantly from that generated during natural HCMV infection [14,15]. Several studies using a guinea pig model have demonstrated that strategies that incorporated gB DNA or, alternatively, subunit vaccines containing recombinant proteins reduced congenital infection and mortality rates [17,18,19]. Recent data further indicate that immunizing mice with gB in its presumed native trimeric or fusion-competent conformation induced markedly higher serum neutralizing antibody titers compared to those elicited by soluble recombinant gB [20,21].

Identifying immunological correlates of protection has been challenging due to the lack of a clear understanding of the mode of action of protective anti-gB antibodies, including their recognition of structures present in the native prefusion conformation of gB on viral particles. A prefusion conformation of glycoprotein B (gB) was recently determined, however, in the absence of gH/gL-containing complexes [22]. Importantly, the conserved heterodimeric gH/gL complex is thought to be essential for triggering the structural reorganization of gB from a fusion-competent but inactive conformation to an active, fusion-competent state [23]. Thus, numerous potential mechanisms have been proposed for the action of anti-gB MAbs, including the following: (i) inhibiting the interaction between gB and the gH/gL complexes, thereby preventing activation of gB [24,25]; (ii) preventing the conformational rearrangement of gB from a fusion-competent prefusion conformation to the postfusion form [22,26,27]; (iii) directly targeting the hydrophobic fusion loops essential for gB’s fusogenic activity [28], among others. Understanding these mechanisms is crucial not only for the generation of potent antiviral antibodies but also for new insights into the structural determinants of gB’s prefusion conformation and the fusion process. 

**Figure 1:**
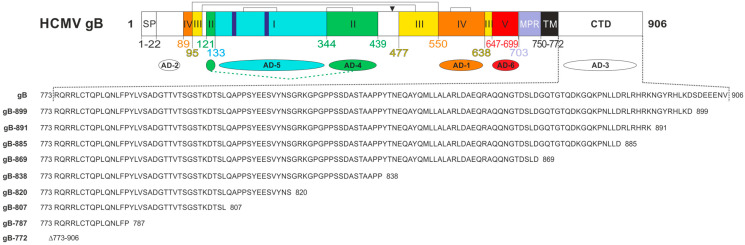
Schematic representation of human cytomegalovirus (HCMV) glycoprotein B (gB)and the gB carboxy-terminal domain (CTD) mutant constructs. (**Top**) Linear representation of full-length HCMV gB, strain AD169 (accession number P06473). The regions representing the structural domains I-V are displayed in different colors in analogy to the crystal structure [27]. Numbers indicate the borders of the individual domains, brackets in the top line indicate disulfide bonds and the arrowhead highlights the cleavage site of the cellular endopeptidase furin. The antigenic domains, named AD-1 through AD-6, are indicated. SP, signal peptide; MPR, membrane proximal region; TM, transmembrane domain; CTD, carboxy-terminal domain. (**Bottom**) Amino acid sequences of the AD169gB CTD (gB) and its truncated derivatives, which were generated and characterized in this study.

Structurally, gB is a homotrimer composed of four distinct regions: a large extraviral ectodomain, a hydrophobic membrane proximal region (MPR), a transmembrane domain (TM), and an intraviral C-terminal domain (CTD) (Figure 1). To date, six antigenic domains (AD-1–6) targeted by gB-specific antibodies have been identified. Among these, all but AD-3 (located at the extreme terminus of the CTD) and the recently discovered AD-6 are capable of inducing neutralizing antibodies during infection [29,30,31]. All characterized neutralizing anti-gB monoclonal antibodies (MAbs) exhibit similar in vitro activity, neutralizing 50% of the input virus at nanomolar concentrations in both fibroblasts and epithelial/endothelial cells [32]. Due to its structural similarities to the vesicular stomatitis virus (VSV) G-protein, HCMV gB is classified as a type III fusion protein [26]. However, unlike VSV-G, HCMV gB is not an autonomous fusogen and requires activation by accompanying protein complexes containing gH/gL [24]. The gB and gH/gL complex is considered as the core fusion machinery, driving the conformational change of gB from the prefusion to the postfusion conformation through mechanisms that are not yet fully understood [22,23,27,33]. 

In a previous study, we demonstrated that the CTD of gB plays a critical role in the regulation of membrane fusion. Specifically, replacing the autologous gB CTD with the CTD of the structurally related fusion protein G of VSV resulted in an intrinsically fusion-active gB variant (gB/VSV-G) [32,34]. Here, we utilized a dual split protein (DSP)-based cell fusion assay to further characterize the determinants of the CTD of gB that are required for the regulation of fusion. To achieve this, we engineered a comprehensive library of gB mutants and identified two derivatives, gB-787 and gB-807, which exhibited autonomous fusion activity in the absence of gH/gL. This fusion activity could be inhibited by all tested gB-specific MAbs and, importantly, was not restricted to MAbs directed against AD-4 or AD-5 as observed with gB/VSV-G. This finding suggests different fusion-active conformations of the two gB variants and, together with previous data, highlights a central role of the CTD in regulating the fusogenicity of HCMV gB.

## 2. Materials and Methods

### 2.1. Oligonucleotides and Expression Constructs 

Oligonucleotides used in this study were purchased from biomers.net GmbH (Ulm, Germany) and are listed in Table 1.

In order to generate the HCMV gB CTD truncation mutants, premature stop codons were introduced via PCR using primer pairs #0–77 and #0–78 to #0–86, respectively. The resulting constructs were named pMN135–pMN143. The potential WW/SH2 motif was mutated by site-directed mutagenesis using primers #5–94 and #5–95 (see Table 1). The expression plasmids coding for HCMV AD169gB and the chimera AD169gB/VSV-G were described elsewhere [32]. The integrity of all newly generated plasmids was confirmed by automated DNA sequence analysis (Macrogen, Amsterdam, The Netherlands). 

### 2.2. Antibodies

The following antibodies were used: the gB-specific human MAbs C23 (Ti23) [35], 1G2 [36], SM10 [36], 2C2 [36], SM5–1 [37], and SDZ 89–104 [38]; the mouse anti-gB MAbs 27–39 [39] and 27–287 [40]; the mouse anti-gH MAb AP86-SA4, referred to as SA4 [41]. The human CMV hyperimmune globulin Cytotect was purchased from Biotest (Dreieich, Germany). The non-neutralizing polyclonal anti-gB serum was isolated from mice immunized with soluble gB (kindly provided by Sanofi Pasteur, Marcy-l’Étoile, France). The naïve mouse serum was purchased from Biozol (Eching, Germany). The anti-β-actin and the Alexa Fluor 555-conjugated secondary antibody for indirect immunofluorescence analyses were both purchased from Thermo Fisher Scientific (Darmstadt, Germany). The horseradish peroxidase-conjugated secondary antibodies for Western blotting were obtained from Dako (Agilent Technologies, Santa Clara, CA, USA).

### 2.3. Cell Culture 

Human embryonic kidney 293T-DSP-mix cells (HEK293T cells stably expressing the dual split proteins DSP1–7 and DSP8–1 co-cultured in a 1:1 ratio [34]) were maintained in Dulbecco’s modified Eagle’s medium (DMEM) (Gibco, Thermo Fisher Scientific, Darmstadt, Germany) supplemented with 10% fetal calf serum (FCS) (Merck, Sigma-Aldrich, Taufkirchen, Germany), glutamine (100 μg/mL), and gentamicin (350 μg/mL). 

### 2.4. Dual Split Protein Cell Membrane Fusion Assay (DSP Assay) 

Development and validation of the DSP assay were described in detail before [34]. Briefly, this assay is based on a pair of chimeric reporter proteins composed of split Renilla luciferase (RLucN and RLucC) and split GFP (GFP1–7 and GFP8–11) which are stably expressed by HEK293T cells termed DSP1–7 (RLucN + GFP1–7) and DSP8–11 (RLucC + GFP8–11) and co-cultured in a 1:1 ratio (293T-DSP-mix cells). Following the expression of a viral fusion protein, cell–cell fusion can be monitored and quantified by the two reconstituted reporter proteins GFP and RLuc. For these assays, 293T-DSP-mix cells were seeded in 24-well (2 × 10^5^ cells/well for GFP-assay) or 96-well plates (3 × 10^4^ cells/well for bioluminescence assay) one day prior to transfection with various gB expression constructs by calcium phosphate precipitation. At 4 h later, cells were washed with phosphate-buffered saline (PBS) and incubated with fresh medium containing 50 µL of diluted mouse serum or 5–25 µg/mL of monoclonal antibody, when indicated. Three days posttransfection, cells were imaged with a CTL Immunospot^®^S6 UV Analyzer (Cellular Technology Limited, Bonn, Germany) and GFP counts were automatically determined using the ImmunoSpot 6.0.0.2 software version (Cellular Technology Limited). For bioluminescence measurement, cells were treated at 48 h posttransfection with 60 µM of the membrane-permeable RLuc substrate EnduRen (Promega, Madison, WI, USA). At 2–24 h later, RLuc activity was measured by using an Orion microplate luminometer (Berthold Technologies, Bad Wildbach, Germany). Experiments were performed at least three times; one representative result is shown. 

### 2.5. cELISA

The cell surface expression of gB was analyzed via cell ELISA (cELISA). For this, 293T-DSP-mix cells (3 × 10^4^ cells/well) were seeded in triplicates (for experimental read-out and background calculations, respectively) in 96-well plates coated with Poly-D-Lysine according to the manufacturer’s protocol (Gibco, Thermo Fisher Scientific, Darmstadt, Germany). At 4–6 h posttransfection, cells were washed with PBS and incubated with fresh medium. After 48 h, cells were washed again once with PBS and incubated for 30 min at 37 °C with anti-gB mouse monoclonal antibody 27–287 (10 µg/mL) diluted in 3% bovine serum albumin (BSA) in PBS for the experimental triplicate set or only 3% BSA/PBS for the control triplicate set. Cells were then washed five times with PBS and fixed for 10 min using a solution of 2% formaldehyde and 0.2% glutaraldehyde (Merck, Sigma-Aldrich, Taufkirchen, Germany) in PBS. Thereafter, cells were washed three times with 3% BSA/PBS and incubated for 30 min at 37 °C with biotinylated goat anti-mouse IgG conjugate (Merck, Sigma-Aldrich, Taufkirchen, Germany) diluted 1:500 in 3% BSA/PBS. Next, cells were washed five times with 3% BSA/PBS before a 30 min incubation with streptavidin–horseradish peroxidase conjugate (Cytivia, Amersham Biosciences Europe GmbH, Freiburg, Germany) diluted 1:10,000 in 3% BSA/PBS. Following five washing steps with PBS-0.1% Tween (Thermo Fisher Scientific, Darmstadt, Germany), 100 µL of tetramethylbenzidine (TMB) peroxidase substrate was mixed 1:1 with peroxidase substrate solution B (KPL) (SeraCare Life Sciences Inc., Milford, MA, USA) and added for 10 min. The reaction was stopped by adding 100 µL of 1 M phosphoric acid. The optical density at 450 nm (OD450) was determined using an Emax microplate reader (Eurofins MWG Operon, Ebersberg, Germany).

### 2.6. Indirect Immunofluorescence Analysis

For indirect immunofluorescence analysis, 293T-DSP-mix cells were grown on coverslips. The next day, cells were transfected via calcium phosphate precipitation. Three days posttransfection, cells were washed two times with PBS before they were fixed with a 4% paraformaldehyde solution for 10 min at room temperature. After three washing steps with PBS, cells were permeabilized with PBS-0.2% Triton X-100 on ice for 20 min. After washing the cells four times with PBS, unspecific binding was blocked by PBS-1% bovine serum albumin (BSA) at room temperature for 30 min. Thereafter, cells were incubated with the anti-gB antibody 27–287 and Alexa Fluor 555-conjugated secondary antibody for 30 min at 37 °C before coverslips were mounted onto microscope slides using DAPI (4′,6-diamidino-2-phenylindole)-containing Vectashield mounting medium (Vector Laboratories, Newark, CA, USA). The samples were analyzed using a Leica TCS SP5 confocal microscope, employing 488 nm and 543 nm laser lines. Each channel was scanned separately under conditions that prevented channel overlap. Representative images were exported and processed with Adobe Photoshop Elements 15, and final image assemblies were created with CorelDraw X6.

### 2.7. Western Blotting

Three days posttransfection, 293T-DSP-mix cells were harvested and lysed by adding sodium dodecyl sulfate polyacrylamide gel electrophoresis (SDS-PAGE) loading buffer, followed by boiling at 95 °C for 10 min and sonication for 1 min. Proteins were separated on 12.5% polyacrylamide gels via SDS-PAGE and subsequently transferred to nitrocellulose membranes (Cytivia, Amersham Biosciences Europe GmbH, Freiburg, Germany). The primary anti-gB antibody C23 was used as a primary antibody and anti-β-actin (Thermo Fisher Scientific, Darmstadt, Germany) served as a loading control. After staining with an appropriate horseradish peroxidase-coupled secondary antibody, ECL-substrate was applied according to instructions of the manufacturer (ECL Western blotting detection kit; Amersham Biosciences Europe GmbH). Chemiluminescence was then detected using an INTAS advanced fluorescence imager (INTAS Science Imaging Instruments GmbH, Göttingen, Germany). 

### 2.8. In Silico Analysis of CTD Sequence and Structure

In order to conduct a sequence conservation analysis, a selection of 447 full-length amino acid sequences was downloaded from the NCBI database (search terms: “Human betaherpesvirus 5 glycoprotein B”, filtered by 900–910 amino acid sequence length). A multiple sequence alignment was performed by using the Clustal Omega tool (https://www.ebi.ac.uk/jdispatcher/msa/clustalo; [42]; accessed on 29 July 2024) and the results were assembled and visualized by using Jalview, Version 2.11.3.3 [43]. AD169 gB (accession number P06473) residues 701–906 were modeled by using AlphaFold2.0 [44]. Structure visualization was performed by using PyMOL (The PyMOL Molecular Graphics System, Version 1.3, Schrödinger, LLC).

### 2.9. Statistical Analysis

Statistical analysis was performed by one-way ANOVA using Bonferroni’s multiple comparison test using GraphPad Prism (version 6; GraphPad Software, USA).

## 3. Results

### 3.1. Generation of Truncation Mutants within the HCMV gB CTD

Previous experiments using a fusion-competent gB/VSV-G chimera suggested a pivotal role of the CTD in the negative regulation of the fusion activity of gB [32]. To further address the relevance of the autologous CTD in controlling gB’s fusion activity, stop codons within the CTD of the gB open reading frame were introduced to generate a comprehensive panel of mutants with progressively truncated CTDs (Figure 1). All mutants were sequence verified and named according to the amino acid (aa) length of the corresponding constructs (gB-899 to gB-772).

### 3.2. Truncation of the CTD Results in Autonomously Fusion-Active gB Variants

**Figure 2:**
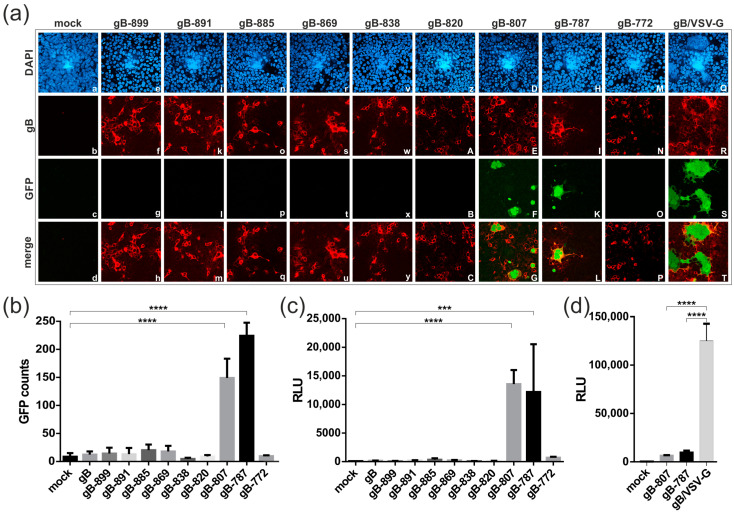
Cell–cell fusion activity of gB CTD-mutants. (**a**–**d**) 293T-DSP-mix cells were transfected with vectors encoding either of the gB constructs shown in Figure 1 or an empty vector (mock), as indicated. The fusion-competent gB/VSV-G chimera served as an internal positive control. (**a**) Indirect immunofluorescence analyses of transfected 293T-DSP-mix cells. Cells were fixed and permeabilized and cell nuclei visualized by DAPI (**top row**). Expression and subcellular protein localization were analyzed via confocal laser scanning microscopy by using the anti-gB antibody 27–287 ((**second row**), gB). Cell–cell fusion was examined via the GFP-signal of the reconstituted DSP-reporter protein ((**third row**), GFP) as well as by the formation of multinucleated syncytia ((**lowest row**), merge). (**b**–**d**) Automated quantification of cell–cell fusion (**b**) via GFP counts at the CTL-Fluorospot reader or (**c**,**d**) by bioluminescence given in relative light units (RLU) upon RLuc-mediated cleavage of EnduRen. Experiments were performed at least three times; one representative result is shown and depicts the mean values of biological triplicates ± standard deviations. Statistical analysis was performed by using one-way ANOVA using Bonferroni’s multiple comparison test, ***: *p* < 0.001, ****: *p* < 0.0001. *p* values refer to cells transfected with mock (**b**,**c**) or gB/VSV-G (**d**) and were not statistically significant if not indicated.

To analyze the membrane fusion capacity of the gB CTD mutants, we took advantage of a well-established dual split protein (DSP) fusion assay [34]. Following gB-induced cell–cell fusion, the activities of the two reporter proteins GFP and Renilla luciferase (RLuc) are reconstituted, allowing for the detection and quantification of the GFP fluorescence and RLuc activity. First, expression of the newly generated constructs was assessed by confocal microscopy, which confirmed comparable expression in 293T-DSP-mix cells transfected with either of the gB constructs (Figure 2a, panels in second row). With respect to membrane fusion capacity, it became apparent that the expression of the CTD mutants gB-807 and gB-787 resulted in the generation of GFP-positive cells with the formation of multinucleated cell syncytia (Figure 2a, third and lowest row). The latter, however, were considerably smaller (factor > 2) than those induced by the hyperfusogenic gB/VSV-G chimera, suggesting a differential degree of fusogenicity of the respective gB variants (Figure 2a; compare panels F and K with panel S).

To remove a potential observer bias from the evaluation of fusion activity, we utilized automated quantification of cell–cell fusions upon complementation of GFP (Figure 2b) or RLuc (Figure 2c,d). In accordance with the confocal images, the two CTD mutants gB-807 and gB-787 induced cell fusions resulting in a significant number of GFP-positive cells (Figure 2b). These findings were further validated by independent experiments upon quantification of RLuc activity, which produced results similar to those obtained from fluorescence-based quantification (Figure 2c). Interestingly, in both assays, the complete deletion of the CTD (Δ773–906) eliminated the fusion function of gB as illustrated by gB-772, similar to the shorter truncation mutants (gB-899 to gB-820), which were non-fusogenic as was observed with the full-length gB (Figure 2a–c), as was demonstrated in earlier studies [34]. As evident from Figure 2d, a quantitative comparison revealed that the fusion activity of the gB/VSV-G chimera [32] was significantly higher than that of the fusion-competent CTD mutants gB-807 and gB-787.

### 3.3. Cellular Localization, Expression Level, and Cell Surface Exposure of the gB CTD Mutants

**Figure 3:**
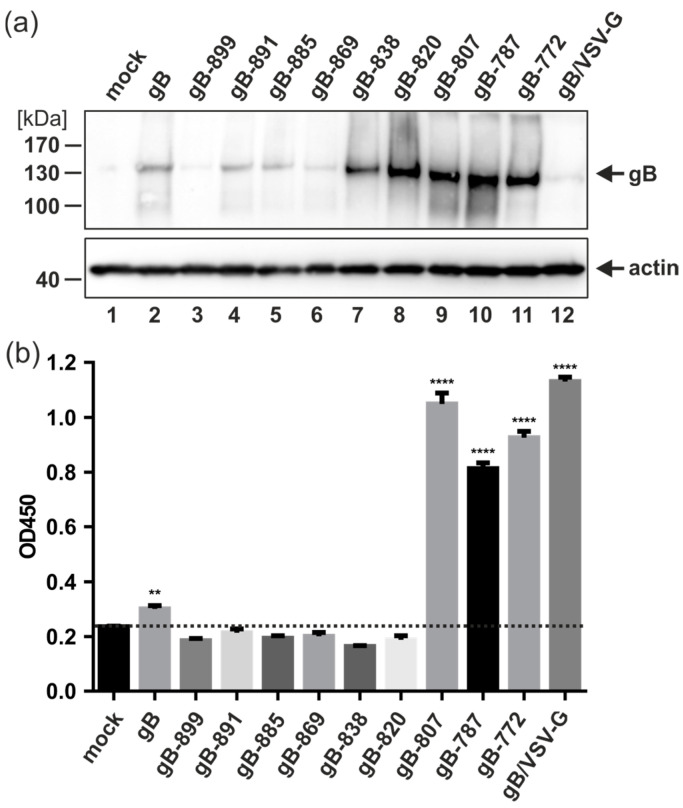
Expression and cell surface exposure of HCMV gB CTD-mutants. (**a**,**b**) 293T-DSP-mix cells were transfected with vectors encoding the indicated gB construct or an empty vector (mock). (**a**) Three days later, Western blot analyses were performed by using anti-gB C23 (upper blot) or β-actin (lower blot) for protein detection. (**b**) Cell surface exposure of gB constructs as evaluated by cELISA. Two days after transfection of triplicates per construct, cells were incubated with the mouse anti-gB antibody 27–287 for 30 min before fixation. Thereafter, cells were washed and incubated with biotinylated goat anti-mouse IgG conjugate followed by incubation with streptavidin–horseradish peroxidase conjugate. Next, TMB peroxidase substrate was added before the reaction was stopped by adding phosphoric acid, and the optical density at 450 nm (OD450) was determined. Experiments were performed at least three times; one representative result is shown. The values depict the mean of biological triplicates ± standard deviations. Statistical analysis was performed by using one-way ANOVA using Bonferroni’s multiple comparison test, **: *p* < 0.01, ****: *p* < 0.0001. *p* values refer to mock transfected cells (dashed line) and were not significantly increased if not indicated.

Confocal microscopy of permeabilized transfected 293T-DSP-mix cells revealed a similar localization pattern for all gB CTD mutants as well as gB/VSV-G, thus arguing against specific cellular localization as an explanation for their distinct fusion capacities (Figure 2a, second row). Next, we examined the total cellular expression of the various gB constructs by Western blotting. As evident from Figure 3a, the mutants starting at gB-838 showed enhanced expression levels as compared to full-length gB (Figure 3a; compare lanes 7 to 11 with lane 2). However, the expression level did not correlate with the cell membrane fusion capacity, as the CTD mutants gB-838, gB-820, and gB-772 were fusion- negative in the DSP assay (see Figure 2). Consistent with these findings, the hyperfusogenic gB/VSV-G variant was expressed at similar levels as the non-fusogenic full-length gB (Figure 3a; compare lanes 12 and 2), indicating that total cellular expression was not correlated with the fusion phenotypes under these experimental conditions.

Lastly, we examined the level of cell surface exposure of gB by cell enzyme-linked immunosorbent assay (cELISA). In agreement with previous studies, the cELISA demonstrated that there was little full-length gB detectable at the cell surface (Figure 3b). The non-fusogenic gB CTD mutants gB-899 to gB-820 exhibited even lower cell surface expression rates which were close to background level (Figure 3b, dashed line). For the fusion-competent gB variants gB-807, gB-787, and gB/VSV-G, significant levels of cell surface expression were detected (Figure 3b). However, there was one notable exception, gB-772 (Δ773–906), which exhibited a high level of cell surface exposure (Figure 3b) but was entirely fusion-negative in the DSP assay (Figure 2). These results indicated that neither the altered cellular localization, the total amount of cellular gB expression, nor the extent of cell surface exposure could be directly correlated with the capacity of gB to induce cell membrane fusions.

### 3.4. Blocking gB-Induced Cell–Cell Fusion by Anti-gB Antibodies

**Figure 4:**
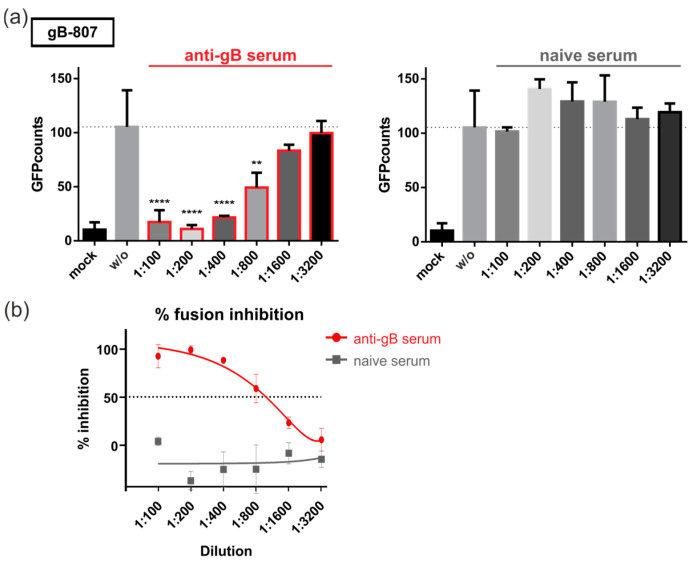
Non-neutralizing polyclonal anti-gB serum blocks gB-807-mediated fusion. (**a**,**b**) 293T-DSP-mix cells were transfected with vectors encoding gB-807 or an empty vector (mock). At 4 h posttransfection, the cultures were washed, and fresh medium was added without serum (w/o) or medium containing log2 dilutions of serum derived from mice immunized with soluble gB (**left**), or naïve serum (**right**), respectively. At 72 h after transfection, cell–cell fusion was quantified by a Fluorospot reader as specified in Materials and Methods. GFP counts were derived from biological triplicates and represent mean values ± standard deviations. Statistical analysis was performed by using one-way ANOVA using Bonferroni’s multiple comparison test, **: *p* < 0.01, ****: *p* < 0.0001. *p* values refer to the gB-807 transfected cells without treatment (w/o, dashed line) and were not statistically significant if not indicated. (**b**) These data from (**a**) were used to calculate the serum-mediated inhibition of fusion in percent based on the GFP counts in relation to the nontreated control (w/o). The half-maximal inhibitory concentration (IC50) is indicated by a dashed line.

**Figure 5:**
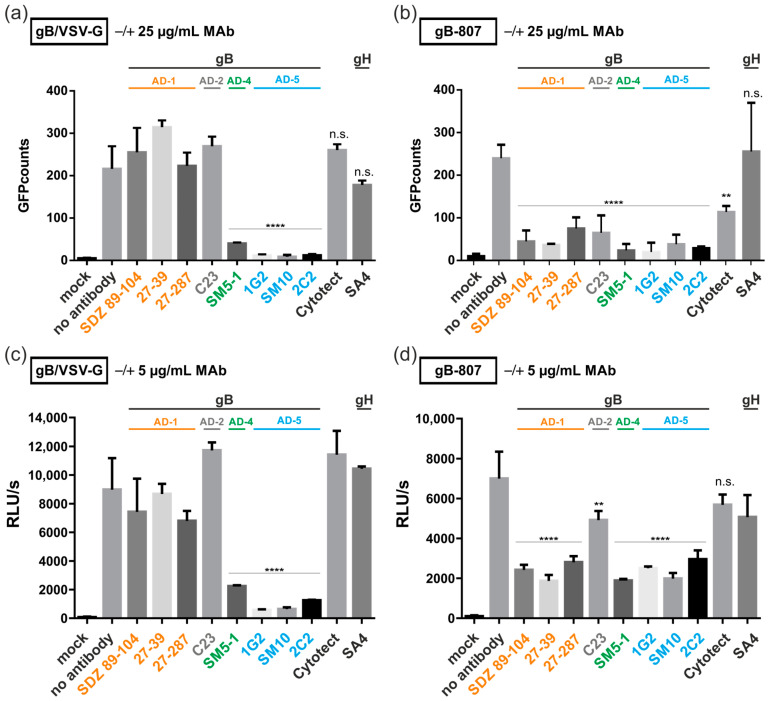
Identification of anti-gB MAbs that potently block membrane fusion of gB/VSV-G or gB-807. (**a**–**d**) 293T-DSP-mix cells were transfected with vectors encoding gB/VSV-G (**a**,**c**), gB-807 (**b**,**d**), or an empty vector (mock). At 4 h posttransfection, the cultures were washed, and fresh medium (no antibody) or medium containing 25 µg/mL (**a**,**b**) or 5 µg/mL (**c**,**d**) of the anti-gB (SDZ 89–104, 27–39, 27–287, C23, SM5–1, 1G2, SM10, or 2C2) and anti-gH (SA4) MAbs as well as 150 µg/mL of the HIG Cytotect was added. At 72 h after transfection, cell–cell fusion was quantified by a Fluorospot reader (**a**,**b**) or by bioluminescence (**c**,**d**) as specified in Materials and Methods. Values were derived from biological triplicates and represent mean values ± standard deviations. Statistical analysis was performed by using one-way ANOVA using Bonferroni’s multiple comparison test, **: *p* < 0.01, ****: *p* < 0.0001. *p* values refer to the transfected cells without treatment (no antibody) and were not statistically significant if not indicated.

We demonstrated recently that gB/VSV-G-mediated fusion was dependent on the ectodomain of gB and could be blocked by monoclonal antibodies directed against gB [32]. Given that all constructs contain the same gB-ectodomain, we next investigated whether their fusion activity could also be inhibited by antibodies. For this, 293T-DSP-mix cells were transfected with fusion-active gB-807 and cultivated in the presence of polyclonal serum (Figure 4a, non-neutralizing anti-gB serum or naïve serum) and monoclonal antibodies (Figure 5), respectively. Three days later, GFP-complementation, as a measure of cell–cell fusion, was quantified automatically. As shown in previous sections, gB-807 yielded in a number of GFP-positive cells, when no serum was added (w/o, dashed lines, Figure 4a). However, upon treatment with anti-gB serum, we observed a dilution-factor-dependent block of cell–cell fusions, which was not observed when serum from naïve mice was added. The half-maximal inhibitory concentration (IC50) of the anti-gB serum was reached at a dilution of 1:1000 (Figure 4b), raising the possibility that non-neutralizing antiviral antibodies could contribute to the inhibition of gB-mediated fusion. 

Having demonstrated fusion inhibition by polyclonal serum, we next addressed the question of whether syncytium formation induced by gB/VSV-G (Figure 5a,c) or gB-807 (Figure 5b,d) could be inhibited by gB-specific monoclonal antibodies. For these experiments, we tested a set of anti-gB MAbs targeting different antigenic domains (AD) of gB (AD-1, SDZ 89–104, 27–39, and 27–287; AD-2, C23; AD-4, SM5–1; AD-5, 1G2, SM10, and 2C2). As controls, we included the human CMV hyperimmune globulin Cytotect as well as an anti-gH MAb (SA4). Cells transfected with an empty vector (mock) or left untreated (no antibody) served as internal controls. Three days after treatment of the transfected 293T-DSP-mix cells with 25 µg/mL (Figure 5a,b) or 5 µg/mL antibody (Figure 5c,d), fusion was quantified by counting GFP-expressing cells (Figure 5a,b) or by RLuc activity (Figure 5c,d). In agreement with our previous report [32], MAbs directed against AD-5 (1G2, SM10, and 2C2) almost completely inhibited cell–cell fusion induced by gB/VSV-G. However, in contrast to our previous publication using gB/VSV-G-GFP as a read-out, the highly sensitive DSP assay employed in this study indicated that the AD-4 specific SM5–1 antibody was equally potent in blocking gB/VSV-G-induced fusion (Figure 5a,c). The polyclonal serum Cytotect was not active in blocking gB/VSV-G even at concentrations of 150 µg/mL (Figure 5a) as was demonstrated in our previous study [32]. In contrast, cell–cell fusions induced by gB-807 were reduced at high concentrations of Cytotect (Figure 5b). Similarly, all anti-gB MAbs used in this experiment significantly reduced the number of syncytia induced by gB-807, at both antibody concentrations. However, the reduction in fusion activity of gB-807 by the anti-AD-2 MAb C23 was slightly lower at lower antibody concentrations (Figure 5b,d). The anti-gH MAb SA4 did not inhibit syncytial cell formation in any of the cells expressing fusion-competent gB/VSV-G or gB-807 (Figure 5). Taken together, we observed a differential capacity of MAbs to inhibit fusion induced by gB/VSV-G compared to gB-807.

### 3.5. C-Terminal Determinants of Regulating gB-Induced Fusion

**Figure 6:**
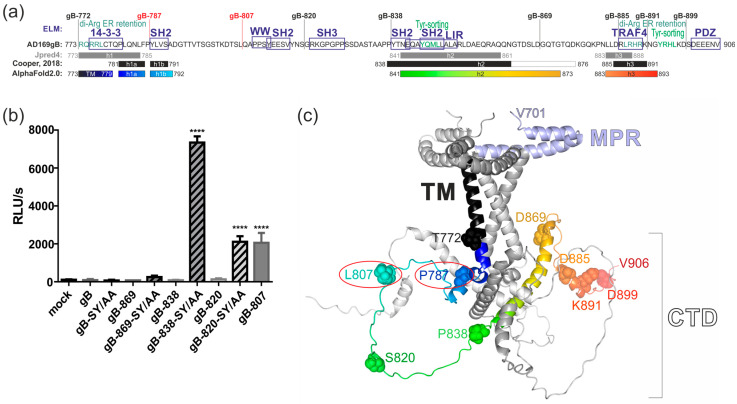
Carboxy-terminal determinants of HCMV gB’s fusion activity. (**a**) The amino acid sequence of AD169-gB is given for its CTD residues 773–906. Vertical lines at the top indicate the truncation mutants used in this study (gB-772 through gB-899; compare Figure 1). The Eukaryotic Linear Motif server (ELM, [45]) was used for the annotation of candidate motifs for interaction with 14–3-3 proteins, Atg8/LC3 (LIR), SH2, SH3, PDZ, TRAF4, or WW domains, as boxed and indicated in purple. Potential sorting motifs are indicated in green (di-Arg-ER retention or Tyr-sorting) and above the sequence. The predicted secondary structures (α-helices h1, h2, and h3) determined by Jpred4 [46] or modeled based on the structure of HSV-1 gB CTD [47] are depicted schematically below the sequence in gray or black, respectively. (**b**) 293T-DSP-mix cells were transfected with the indicated wild-type constructs of gB, gB-869, gB-838, and gB-820 or their mutated derivatives containing the amino acid substitutions S812A+Y813A (SY/AA). Cells transfected with an empty vector (mock) or the fusion-competent gB-807 served as internal negative and positive controls, respectively. Cell–cell fusion was quantified by bioluminescence as described in Materials and Methods section at 72 h posttransfection. Statistical analysis was performed by using one-way ANOVA using Bonferroni’s multiple comparison test, ****: *p* < 0.0001. *p* values refer to cells transfected with mock and were not statistically significant if not indicated. (**a**,**c**) Residues 701–906 of AD169gB were modeled by AlphaFold2.0 [44]. The results for the CTD are indicated schematically in (**a**) and the obtained structure is highlighted in (**c**) for one of the trimeric gB as follows: MPR is in light blue, TM is in black, and the CTD is rainbow colored from the N-terminus (dark blue) to C-terminus (dark red). The two other monomers of the gB trimer are depicted in gray. The terminal residues of the different truncation variants are shown in space-filled presentation and are labeled. The termini of the intrinsically fusion-competent gB-787 or gB-807 are highlighted and circled in red.

Together, our results led us to hypothesize that substitution or truncation of the gB C-terminus may disrupt an interaction between gB and a cellular protein, thereby triggering fusion. We therefore analyzed the gB C-terminus for short linear protein–protein interaction motifs by using the Eukaryotic Linear Motif (ELM)-server (http://elm.eu.org/, accessed on 25 May 2024) [45]. The results of the predictions are presented in Figure 6a and indicate that the presence of TRAF4- or PDZ-interaction motifs in the C-terminal-most 21 residues of gB did not influence protein expression level, cell surface exposure, or fusogenicity (Figure 2 and Figure 3). The deletion of residues 839–869 resulted in significantly higher protein expression (Figure 3a; compare lanes 6 and 7), suggesting that the presence of the two SH2-binding motifs and/or the autophagy-related LC3 interaction region (LIR) immediately downstream of residue 838 negatively impact gB protein expression, although this did not affect the construct’s capacity to induce cell–cell fusion (Figure 2). Likewise, the deletion of potential sorting motifs, such as di-Arginine-ER retention or Tyrosine-sorting signals, showed no apparent impact on cell surface exposure. Specifically, the gB-838 variant, which lacks three predicted sorting motifs, did not exhibit enhanced surface exposure compared to gB-820 or the fusion-competent constructs gB-807 and gB-787 (Figure 3b). However, further truncation, as demonstrated for gB-807 and shorter constructs, resulted in significantly increased cell surface expression. This result suggested a critical regulatory role of potential SH2/WW-interaction motifs (aa 810–817) in modulating cell surface presentation and fusion activity (Figure 2 and Figure 3b). Notably, mutating the respective SH2/WW-interaction motifs by substituting S812 and Y813 with alanine did not confer fusion-competence to full-length gB or gB-869. In contrast, these same substitutions rendered gB-838 and gB-820 fusion-active (Figure 6b). These findings suggest that the enhanced fusion activity observed following deletions or mutagenesis within the gB-CTD may only partially be attributed to the loss of interactions with cellular WW/SH2-domain-containing proteins.

As a result of these findings, we conducted a secondary structure prediction using Jpred4 (https://www.compbio.dundee.ac.uk/jpred/; accessed on 25 May 2024) [46]. This analysis revealed three alpha helical structures (h1, h2, and h3) that are almost congruent with the previously determined structure for the gB-CTD of the alphaherpesvirus HSV-1 [47] (Figure 6a; compare gray and black lines). Since the intracellular region is not resolved in the experimental structures of HCMV gB [22,27], we utilized AlphaFold2.0 to model AD169gB residues 701–906, encompassing the membrane proximal region (MPR), transmembrane (TM), and carboxy-terminal domain (CTD) (Figure 6a,c). The predicted tertiary structure indicates that HCMV gB forms an intertwined trimer, with the two MPR helices located on the outer layer of the plasma membrane, which is spanned by the transmembrane domain. Notably, the TM helix of HCMV gB extends from amino acid 772 to residue 779, consistent with the crystal structure of alphaherpesviral HSV-1 gB [47]. The first intraviral/intracellular helix 1 initiates at proline 781, a residue well conserved across all herpesvirus subgroups [47]. This helix is relatively short (h1, residues 781–792) and lies beneath the plasma membrane. Helix 1 is followed by a long unstructured segment and helix 2 (h2, residues 841–873), which forms various contacts with the neighboring helices of the other two gB monomers. The CTD is terminated by shorter helix 3 (h3, residues 883–893). 

Based on the results from the DSP assays and the modeled structural data, it appeared that all fusion-active gB constructs required at least the intraviral/intracellular helix 1a, likely to anchor the gB ectodomain during the fusion process. This is supported by the observation that truncation of the TM at residue 772 results in fusion-defective gB (Figure 2). It could be argued that residues 808–906 were crucial for maintaining the fusion-competent but inactive state of the gB ectodomain, since the deletion of this region, as seen in gB-807 and gB-787, resulted in intrinsically fusion-competent gB. A dominant role for fusion regulation can then be attributed to the CTD h2, which forms multiple inter-gB interactions to maintain the CTD trimer. An additional layer of fusion regulation is facilitated by the recruitment of cellular SH2/WW-domain-containing proteins to residues 810–817. However, the recruitment of cellular protein(s) to this motif is not sufficient for fusion and becomes apparent only after the retrieval of fusion-inhibiting activity provided by the CTD h2 (Figure 6b).

## 4. Discussion

The viral fusogen HCMV gB is crucial for viral infection, facilitating both the entry and cell-to-cell transmission of intracellular virions through the formation of multinucleated cell syncytia. These syncytia have been observed in the tissues of HCMV-infected individuals and in vitro following the inoculation of diverse cell types with various clinical isolates or lab strains of HCMV [48,49,50,51,52,53,54,55]. Given gB’s essential role in viral infectivity and spread, as well as its immunodominance in eliciting adaptive immune responses, gB has been identified as an obvious target for prophylactic vaccine development. Multiple vaccine candidates incorporating gB have undergone early clinical trials [56,57,58]. However, analyses of the adaptive immune responses elicited by these candidates indicate a need for a more comprehensive understanding of the immune response to gB in order to develop vaccines that can elicit enhanced protective antibody responses [15,16,31,59]. Here, we applied a virus-free DSP cell fusion assay to characterize the determinants in the C-terminus of gB required for fusion activity. We identified the two truncation mutants, gB-787 and gB-807, which demonstrated cell–cell fusion capabilities. However, the extent of cell fusion mediated by these constructs was significantly lower compared to the gB/VSV-G construct (Figure 2). Of note, neither the cellular expression level nor the degree of cell surface exposure of gB directly correlated with the fusion activity (Figure 3), suggesting other properties to be responsible for the observed fusion phenotypes. The findings of this study demonstrate for the first time that the CTD of betaherpesviral HCMV gB has a critical role in regulating the fusion activity of this viral glycoprotein, analogous to its homologs in alpha- and gammaherpesviruses. Previous studies have shown that truncations, point mutations, or insertions in the CTDs of gB homologs can induce hyperfusogenic phenotypes in Herpes Simplex Virus (HSV)-1, HSV-2, Varicella-Zoster Virus (VZV), Pseudorabies virus (PrV), Epstein–Barr virus (EBV), and Human herpesvirus 8 (HHV-8) infections ([60,61] and references therein). In agreement with our findings, the fusion activity of gB-homologs from HSV-2, EBV, and HHV-8 does not correlate with gB’s protein expression or surface exposure [62,63,64]. Despite these similarities, significant differences exist in the roles of CTDs in the fusion regulation of gBs encoded by various herpesviruses. This is illustrated by findings that neither the mutation of the predicted HCMV gB sorting motifs (gB-mutEndo, Y845A + LL883/884AA + Y894A) nor the exchange of the TM and CTD of HCMV gB with those of the hyperfusogenic HSV-1 LL871/872 gB variant (gB-HSVmutLL) resulted in a fusogenic form of HCMV gB [32]. All of these mutants were also negative in this DSP assay (Reuter and Kropff, unpublished). We also did not identify any positively charged lysine cluster or canonical immunoreceptor tyrosine-based inhibition motif (ITIM) consensus sequence, [ILV]XYXX[LV], within the CTD of HCMV gB (Figure 6a). These motifs have been shown to act as negative regulators of syncytial phenotypes induced by VZV gB [65,66], but cannot be found within the CTD of HCMV gB. To further explore the regulation of the membrane fusion activity of HCMV, we conducted a web-based ELM prediction analysis, which indicated that residues 810–817 could potentially contain critical protein–protein interaction motifs (see Figure 6a). Interestingly, disrupting these SH2/WW-interaction motifs affected gB’s cell-surface presentation and fusion capacity only in the absence of CTD helix 2 (gB-838, gB-820, and gB-807), but not when it was present (gB and gB-869) (Figure 3b and Figure 6b). 

These findings suggest that the regulation of HCMV gB fusion activity is primarily structural rather than dependent on protein–protein interactions. Consistently, only chimeric constructs in which the MPR, TM, and large CTD of gB were replaced with the TM domain and/or the unstructured and short CTD of VSV-G or baculovirus gp64 exhibited constitutive membrane fusion capacity ([20,32] and Figure 2). Secondary structure predictions, combined with AlphaFold2.0 modeling, identified a TM helix spanning amino acids 750 to 779, which is notably longer than the TM helix (i.e., aa 750–772) predicted by the TMHMM server (https://services.healthtech.dtu.dk/services/TMHMM-2.0/; accessed on 30 June 2024). The fusion-negative gB-772 mutant, localized to the cell surface (Figure 3b), supports previous findings that hydrophobic residues (aa 751–771) are essential and sufficient for membrane anchoring [67], yet insufficient for inducing membrane fusion (Figure 2). It is likely that a longer TM helix is necessary to position the MPR on the outer layer and CTD helix 1 on the inner layer of the plasma membrane at an appropriate distance during fusion. This positioning appears crucial for anchoring the gB ectodomain during the fusion process, as the deletion of the extended TM and helix 1, as seen in the non-fusogenic gB-772 mutant, results in the loss of fusion activity (Figure 2). The HCMV gB CTD helix 2 appears to function as the primary negative regulator of gB fusion, as the deletion of this helix enables the ectodomain to induce cell–cell fusion (Figure 2). Given its multiple connections with neighboring gB-monomers, it is plausible that helix 2 plays a crucial role in maintaining the trimeric CTD structure (Figure 6c). This hypothesis is supported by extensive studies on truncation and amino acid substitution mutants of HSV-1 and HSV-2 gBs [47]. In these studies, helix 2 is essential for maintaining the trimeric CTD structure, as hyperfusogenic mutations often target residues at the trimeric CTD interfaces, disrupting interprotomer bonds such as hydrogen bonds, van der Waals contacts, and hydrophobic interactions or indirectly destabilizing trimeric interfaces by altering important structural elements and helix 2 [47,68]. Thus, it is plausible that CTD h2 is crucial for maintaining HCMV gB in its trimeric conformation, acting as the primary negative regulator of fusion. Upon deletion of h2, the recruitment of cellular interaction partners to the WW/SH2-interaction motif likely establishes a secondary negative regulatory mechanism to prevent membrane fusion. These hypotheses are supported by the observation that constructs containing h2 but no WW/SH2 motif were fusion-incompetent, in contrast to constructs lacking both h2 and the WW/SH2 motif (Figure 6b). This suggests that (i) the N-terminal fragment of h2 comprising residues 839–869 is sufficient for the negative regulation of gB’s fusion activity; and (ii) the recruitment of WW/SH2-domain-containing proteins is secondary to structural constraints. Likely, the WW-domain-containing members of the Nedd4 family of ubiquitin E3 ligases, which were demonstrated to bind to gB’s C-terminal PPXY motif, are involved in the regulation of fusion activity [69]. Given the CTD’s significant role in fusion regulation, we analyzed its evolutionary conservation by conducting a multiple sequence alignment of 447 published full-length HCMV gB sequences using the Clustal Omega tool [42]. This analysis demonstrated a high degree of amino acid conservation within HCMV gB CTD (aa 773–906) across the sequences examined, with 90.3% sequence similarity and 81.3% sequence identity (Supplementary Materials Figure S1). The high degree of conservation observed in the SH2/WW-interaction motifs (aa 810–817) and the N-terminal part of h2 (aa 839–865) likely underscores their critical roles in regulating gB’s fusion activity. However, we identified twenty-six polymorphisms, primarily located between CTD helices h1 and h2 (eleven polymorphisms), within the C-terminal part of h2 (aa 866–873, six polymorphisms) or downstream of h3 (six polymorphisms), while h1 and h3 are 100% conserved (Supplementary Figure S1). Future research should investigate whether and how these CTD polymorphisms influence gB’s fusion activity and the pathogenicity of different HCMV strains. 

Despite the shared critical role of gB-CTDs across all herpesvirus subfamilies in fusion regulation, they appear to be differentially regulated. Mutational analysis of EBV gB has revealed two distinct domains within its CTD that function differentially in the fusion process. While EBV CTD helix 2 was necessary for productive membrane fusion, helix 3 and/or the very C-terminal end of the protein both act as negative regulators of membrane fusion [63,70]. Similarly, partial loss of the terminal helix 3 is sufficient to generate syncytial phenotypes in HSV-1/2 gBs. In contrast, CTD helix 3 as well as the very C-terminal end were dispensable for the autonomous fusogenicity of HCMV gB (Figure 2). Furthermore, the complete loss of h1b, h2, and h3 resulted in non-fusogenic variants of HSV-1 gB but led to hyperfusogenic activity in HCMV gB-787 and gB-807 ([47,68] and Figure 2). One important limitation of the present study is that we obtained results for fusion-competent gB-807 and gB-787 in the absence of gH/gL. This contrasts with the above-mentioned results for HSV-1/2 and EBV, which were derived primarily from infected cells or cells expressing gB in combination with the full set of gH/gL-containing complexes, which are believed to trigger gB’s fusion activity in the context of virus infection. In pre- and postfusion states, gB forms stable complexes with gH/gL through multiple interactions of their respective ectodomains and CTDs [71,72]. It is hypothesized that upon receptor binding, the gH-CTD inserts like a wedge into the surface pocket formed by the trimeric gB-CTD, releasing the inhibitory clamp [73]. Given the structural conservation of gB and gH across all herpesviruses, this activation mechanism might also be used by other gB orthologs. Thus, it is conceivable that the deletion of CTD h2 in gB-787 and gB-807 similarly relieves an inhibitory clamp, and induces a structural reorganization of the ectodomain that ultimately initiates gB’s fusion process in the absence of gH/gL. Future work will focus on determining whether and how the presence of gH/gL affects the fusogenicity of gB truncation and amino acid exchange mutants. However, this was not the scope of the current study, which aimed to establish a cell–cell fusion assay to identify the C-terminal determinants of gB’s fusion regulation and compare the mode of action of anti-gB antibodies in the absence of gH/gL.

The DSP assay revealed that, contrary to our previous publication, gB/VSV-G-mediated membrane fusion could be efficiently inhibited by MAbs directed not only against AD-5 but also against AD-4 of gB (Figure 5). This discrepancy is likely attributable to the higher sensitivity of the DSP assay, which does not require de novo protein expression of the reporter proteins GFP and RLuc. Additionally, the GFP moiety, which was fused to the C-terminus of gB/VSV-G for automated quantification in our previous study, may have affected the structure of the gB-ectodomain, limiting the access of AD-4 specific antibodies to this construct [32]. Interestingly, the fusion capacity of the CTD mutants gB-807 and gB-787 was blocked by all tested antibodies, irrespective of whether they were neutralizing or non-neutralizing (Figure 5). This might be explained by the virus-free nature of the DSP assay, which allows binding and fusion inhibition by antibodies that fail to neutralize the authentic virus containing gH/gL-complexes, which shield the corresponding target structures on HCMV-gB. Intriguingly, the observation that the autonomous fusion activity of gB/VSV-G, in contrast to that of the CTD mutants gB-807 and gB-787, can only be inhibited by a subset of anti-gB antibodies suggests that these gB variants exist in different fusion-competent conformational states. Since only MAbs targeting AD-4 and AD-5, which bind directly next to or near the viral fusion loops, can block gB/VSV-G-induced fusions, this may indicate that gB/VSV-G has already transitioned to a conformational state where the fusion loops are exposed, such as the extended intermediate state of gB prior to membrane fusion [22]. Consequently, antibodies targeting conformational rearrangements of gB prior to this intermediate state may no longer be functional for gB/VSV-G but may still be active against the fusogenic CTD mutants. Alternatively, it is possible that the non-native VSV-G part of the chimera impacts the tertiary structure of gB’s ectodomain. However, this explanation is unlikely, as all antibodies utilized in this study, which target different antigenic epitopes within gB’s ectodomain, have been shown to efficiently bind to the gB/VSV-G derivative [32]. In support of this argument, no significant conformational differences in the ectodomain of hyperfusogenic CTD mutants compared to wild-type gB were observed when using a comparable panel of conformational antibodies specific to different ectodomain epitopes of HSV-1 gB [68].

Previous vaccine candidates, including live attenuated or subunit adjuvanted platforms based on monomeric gB, have had only limited success and are generally considered to exhibit similarly limited immunogenicity [74]. To address this, novel vaccine approaches have been developed. As an example, enveloped virus-like particles (eVLPs) incorporating membrane-bound chimeric gB/VSV-G induced a neutralizing antibody response in mice that was 10-fold higher compared to their soluble recombinant protein counterpart [20]. This eVLP vaccine has progressed to phase I clinical studies in HCMV-seronegative individuals, confirming its safety and immunogenicity [75]. Additionally, the mRNA-1674 vaccine encoding gB and the pentameric complex has been shown to be safe and to produce long-lasting immune responses against various HCMV strains in another phase I trial [76,77]. However, it will be essential to determine whether these vaccines induce sufficient quantities of fusion-inhibiting antibodies. The current study suggests that MAbs directed against AD-4 and AD-5 would be particularly desirable, as they can inhibit the cell–cell fusion activity of both fusion-competent gB variants, gB/VSV-G as well as gB-807 and gB-787. The newly developed DSP assay represents a valuable tool for selective screening of MAbs and polyvalent antisera that efficiently interfere with gB-mediated fusion, potentially leading to more effective HCMV immunotherapeutic agents.

## Figures and Tables

**Table 1:** Primers used for cloning or site-directed mutagenesis.

Primer No.	Name	Sequence
0–77	5EcoR1-AD169gBcoopt	GCATGAATTCAACTCCTACAAGCAGCGCG
0–78	3Xho-AD169gBcoopt899STOP	GCATCTCGAGTCAGTCCTTCAGGTGCCGGTAGCC
0–79	3Xho-AD169gBcoopt891STOP	GCATCTCGAGTCACTTTCTGTGCCGCAGCCGGTCC
0–80	3Xho-AD169gBcoopt885STOP	GCATCTCGAGTCAGTCCAGCAGGTTGGGCTTCTGGCCC
0–81	3Xho-AD169gBcoopt869STOP	GCATCTCGAGTCAATCCAGGCTGTCGGTGCCATTCTG
0–82	3Xho-AD169gBcoopt838STOP	GCATCTCGAGTCAGGGTGGGGCGGCTGTAGAGGCATC
0–83	3Xho-AD169gBcoopt820STOP	GCATCTCGAGTCAGCTGTTGTACACGGACTCTTCGTAGC
0–84	3Xho-AD169gBcoopt807STOP	GCATCTCGAGTCACAGGGAGGTATCCTTGGTGCTGC
0–85	3Xho-AD169gBcoopt787STOP	GCATCTCGAGTCAAGGGAACAGGTTCTGCAGGGGCTG
0–86	3Xho-AD169gBcoopt772STOP	GCATCTCGAGTCAGGTGTAGATCAGGTATGTGATGATCACG
5–94	5ADgBcoop S812A+Y813A	GCAGGCCCCACCCGCCGCCGAAGAGTCCGTGTA
5–95	3ADgBcoop S812A+Y813A	TACACGGACTCTTCGGCGGCGGGTGGGGCCTGC

## Data Availability

The original contributions presented in the study are included in the article/Supplementary Material; further inquiries can be directed to the corresponding author.

## Supplementary Materials

The following supporting information can be downloaded at: https://www.mdpi.com/article/10.3390/v16091482/s1, Figure S1: Conservation of the HCMV gB CTD.
