# Supplementary material for: The Autonomous Fusion Activity of Human Cytomegalovirus Glycoprotein B Is Regulated by Its Carboxy-Terminal Domain
Source: Viruses. 2024 Sep 18;16(9):1482. doi: 10.3390/v16091482 (PMC11437439; doi:10.3390/v16091482)
Supplement: Supplementary file 1 [file viruses-16-01482-s001.zip › viruses-3170505-supplementary.pdf]

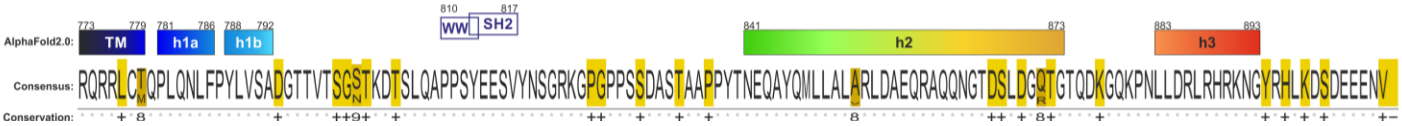

Figure S1. Conservation of the HCMV gB CTD. Summary of the multiple sequence alignment of 447 gB sequences from the NCBI database which was assembled as described in the Materials and Methods section. Conservation is visualized as a histogram within the consensus sequence and given below as a numerical index reflecting the conservation of physico-chemical properties in the alignment: Identities score highest (score of 11, colorless and indicated by a '*'), and the next most conserved group contains substitutions, where all properties are conserved (score of 10, highlighted and marked with a '+'). At the top, the AlphaFold2.0-modeled CTD (h1, h2 and h3) is given rainbow colored from N-terminus (dark blue) to C-terminus (dark red), and purple boxes depict linear motifs for interaction with proteins containing WW/SH2 domains as predicted by the ELM server (compare Figure 6).
